# Postoperative complications in Hartmann’s procedure *versus* intersphincteric abdominoperineal excision in rectal cancer: randomized clinical trial (HAPIrect)

**DOI:** 10.1093/bjsopen/zraf093

**Published:** 2025-09-30

**Authors:** Maziar Nikberg, Viktor Åkerlund, Torbjörn Swartling, Pamela Buchwald, Kenneth Smedh, HAPIrect Collaborative Study Group, Eva Angenete, Helgi Birgisson, Abbas Chabok, George Dafnis, Markku Haapamäki, Peter Matthiessen, Pär Myrelid, Gert Nestler, Christoffer Odensten, Jukka Rintala, Thorbjörn Sakari, Josefin Segelman, Ingvar Sverrisson, Niklas Zar, Philippe Wagner

**Affiliations:** Department of Surgery, Västmanland's Hospital Västerås, Västerås, Sweden; Centre for Clinical Research Region Västmanland, Uppsala University, Västerås, Sweden; Department of Surgery, Västmanland's Hospital Västerås, Västerås, Sweden; Centre for Clinical Research Region Västmanland, Uppsala University, Västerås, Sweden; Department of Surgery, Institute of Clinical Sciences, Sahlgrenska Academy, University of Gothenburg, Sahlgrenska University Hospital/Östra, Gothenburg, Sweden; Department of Surgery, Skåne University Hospital, Malmö, Sweden; Department of Clinical Sciences, Malmö, Lund University, Malmö, Sweden; Department of Surgery, Västmanland's Hospital Västerås, Västerås, Sweden; Centre for Clinical Research Region Västmanland, Uppsala University, Västerås, Sweden

**Keywords:** lower gastrointestinal surgery

## Abstract

**Background:**

In patients with rectal cancer, when it is not possible to restore bowel continuity with an anastomosis, the optimal surgical method is still a matter of debate. The aim of this trial was to determine 30-day postoperative surgical complication rates after Hartmann’s procedure (HP) *versus* intersphincteric abdominoperineal excision (iAPE) in patients with rectal cancer who were not suitable for restorative surgery.

**Methods:**

This multicentre randomized controlled trial (HAPIrect) was performed in Sweden and Finland between 2014 and 2021. Eligible patients with adenocarcinoma of the rectum located ≥ 5 cm from the anal verge and deemed unsuitable for anterior resection with anastomosis were randomized (1:1) intraoperatively to either HP or iAPE. The primary outcome was 30-day postoperative surgical complications. Secondary outcomes were major surgical complications (Clavien–Dindo grade ≥ IIIa), perineopelvic complications, and overall complications. Logistic regression in the intention-to-treat population was the primary method used to compare the surgical approaches.

**Results:**

Of 194 eligible patients, 163 were randomized (80 patients to HP and 83 to iAPE). The study was closed before achieving the target accrual. The main reasons for not receiving an anastomosis were advanced age, co-morbidity, or poor anal sphincter function. Mean operating time in the HP and iAPE groups was 291 and 373 minutes, respectively. In the HP and iAPE groups, the surgical complication rate was 39% and 43%, respectively (odds ratio (OR) for HP 0.83; 95% confidence interval (c.i.) 0.44 to 1.54; *P* = 0.549) and the rate of major surgical complications was 14% and 11%, respectively (*P* = 0.573). Perineopelvic complications occurred in 21% and 30% of patients in the HP and iAPE groups, respectively (OR for HP 0.63; 95% c.i. 0.31 to 1.28; *P* = 0.197). The overall complication rate (including both medical and surgical complications) was 45% and 49% in the HP and iAPE groups, respectively (*P* = 0.574). In multivariable analysis adjusted for sex, preoperative radiotherapy, and surgical procedure, there was no statistically significant difference in surgical complications between the two groups.

**Conclusion:**

Although the trial was underpowered and did not reach accrual, in randomized patients, both HP and iAPE are practicable surgical options for patients unsuitable for anastomosis.

**Registration number:**

NCT01995396 (http://www.clinicaltrials.gov).

## Teaser

Both Hartmann’s procedure and intersphincteric abdominoperineal excision are viable surgical options for patients unsuitable for anastomosis. Surgeons should use the technique in they are most confident with and experienced in.

## Introduction

Low anterior resection (LAR) with total mesorectal excision is the standard in rectal cancer treatment^1^. However, not all patients are suitable candidates for LAR, particularly those with poor anal sphincter function, because of an increased likelihood of postoperative dysfunction, such as incontinence and LAR syndrome, that could significantly affect patients’ quality of life^2^. Other factors making patients poor candidates for anastomosis include advanced age and frailty, co-morbidities, and performance status, with the risk of anastomotic leakage and other serious adverse outcomes.

In Sweden, there has been a notable increase in the use of Hartmann’s procedure (HP) for rectal cancer, rising from less than 5% in the mid-1990s to approximately 15% more recently^3^. The most pronounced increase has been observed among patients with metastatic rectal cancer, reaching rates of 30%^4,5^, probably in order to avoid an anastomotic leak and compromise oncological therapy.

HP is regarded as safe by many surgeons, and is a straightforward and relatively quick operation with a low incidence of major complications and mortality^6–10^. However, some studies^11–13^ have reported high complication rates following HP, especially when the rectum is transected at a low level, resulting in pelvic abscess frequencies of 11–33%. There are also potential functional disorders from the anorectal stump, such as secretion and or bleeding. An alternative to HP when LAR is not feasible is intersphincteric abdominoperineal excision (iAPE), which preserves the external anal sphincter and the muscular pelvic floor. This approach results in a smaller perineal defect, enabling tension-free muscular closure compared with the traditional abdominoperineal excision (APE) with sutures in the fatty tissues. There is also no need for a perineal mesh or muscle flap with iAPE. Thus, iAPE could lead to fewer postoperative complications, no functional problems from the anorectal stump, and potentially improve long-term quality of life. Nonetheless, it does have some disadvantages, such as increased surgical trauma, the need for a Lloyd–Davis lithotomy position and associated risks, and a longer operative time.

The existing literature comparing postoperative outcomes after rectal cancer surgery with HP *versus* iAPE is inconclusive. Two meta-analyses, mainly based on small retrospective studies, resulted in conflicting conclusions. Although one meta-analysis reported no significant difference in postoperative complications between the two procedures^14^, the other suggested a lower complication risk with iAPE^15^. Furthermore, a recent non-randomized prospective study from the UK^16^ reported comparable surgical complication rates for HP and iAPE. To date, the largest registry-based cohort study conducted in Sweden^17^ has indicated a higher risk of surgical complications after HP.

The aim of the present multicentre randomized clinical trial (RCT) was to compare the 30-day postoperative surgical complication rate after HP *versus* iAPE. The hypothesis was that iAPE, compared with HP, would be associated with a reduced incidence of postoperative complications.

## Methods

### Study design

The HAPIrect study was a multicentre RCT conducted across 14 Swedish and 1 Finnish hospital. The HAPIrect study was conducted in accordance with the Declaration of Helsinki guidelines and was approved by the Swedish Ethical Review Board in Uppsala (No. 2013/297) and the Regional Medical Research Ethics Committee of North Ostrobothnia, Finland (EETTMK: 93/2018). Reporting was according to the CONSORT statement.

This trial compared postoperative surgical complications between HP and iAPE in patients diagnosed with rectal cancer. The preoperative assessment for electively scheduled adult patients included routine demographic data, age, sex, co-morbidities, World Health Organization (WHO) performance status, American Society of Anesthesiologists (ASA) grade, body mass index (BMI), smoking habits, preoperative radiotherapy, reasons for not performing LAR, tumour height, and the Tumour Node Metastases (TNM) stage. General clinical assessment, such as digital rectal examination and proctosigmoidoscopy, was performed and additional diagnostic evaluations included colonic examination, abdominothoracic computed tomography scans, and magnetic resonance imaging of the rectum. Eligible patients were those aged ≥ 18 years diagnosed with adenocarcinoma of the rectum located 5–15 cm from the anal verge in whom restorative surgery was not planned (*Table S1*). The decision to perform non-restorative surgery was based on the attending surgeon's clinical experience, patient preferences, and variables such as old age, co-morbidities, and/or weak sphincter function and overall health.

**Fig. 1:**
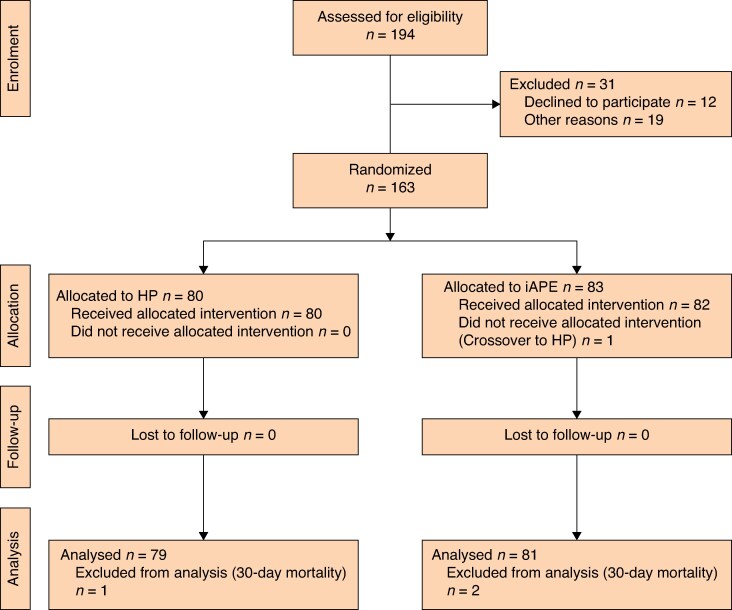
CONSORT flow chart for HAPIrect trial HP, Hartmann’s procedure; iAPE, intersphincteric abdominoperineal excision.

Written informed consent was obtained from patients before surgery. The initials of patients who declined to participate in the study, as well as their reasons for non-participation, were documented (*Fig. 1*). The study enrolled from December 2014 and was concluded in December 2021.

### Randomization

To reduce selection bias for either surgical procedure, consented patients were randomized during the surgery after verifying that both procedures could be performed and after completion of the mesorectal excision. The randomization sequence was centralized and generated through a web-based platform stratified by hospital, with a block size varying between four and six. Importantly, the operating surgeon was not aware of the assigned surgical method (HP or iAPE) until the time of randomization during the surgery.

### Participating hospitals and surgical technique

Hospitals joined the study at different times starting from December 2014. Standard surgical protocols according to local practice were followed, including antibiotic and thrombosis prophylaxis, continuous thoracic epidural analgesia, and the use of laxatives. Surgical procedures were performed by total mesorectal excision surgeons skilled in both HP and iAPE, as stated by the study protocol. Abdominal dissection was performed using either open or minimally invasive (laparoscopic/robotic-assisted) surgery, with either high or low ligation of the inferior mesenteric artery. Total mesorectal excision was performed down to the pelvic floor. A bowel clamp or linear stapling device was applied below the tumour, followed by a rectal washout as per local practices.

In patients randomized to HP, the stapler was fired, removing the tumour-bearing section, and the distal colon was exteriorized as an ostomy. For patients undergoing iAPE, the approach varied depending on the surgeon’s preference. In laparoscopic surgery, the bowel was cut while in open surgery the stapler was either fired without cutting the bowel or fired and the tumour-bearing section removed before perineal dissection. The anus was sealed with two purse-string sutures and the dissection was performed in the intersphincteric plane medial to the external sphincter, levator muscle, and puborectalis sling into the pelvis. The specimen was extracted either perineally or abdominally after minimally invasive surgery, usually through a Pfannenstiel incision. The perineal defect was closed with sutures in the puborectalis, the levator, and the external anal sphincter. One or two abdominal drains into the pelvis were recommended. There was no recommendation to fill the pelvis. A more detailed procedural description is available in the original HAPIrect trial study protocol^18^.

### Follow-up

Consistent with the HAPIrect trial protocol, follow-up evaluations were conducted 30 days after surgery by surgeons in the colorectal teams to assess complications, with data recorded prospectively in study-specific and Swedish Colorectal Cancer Registry recording forms.

### Outcomes of interest

The primary endpoint was 30-day surgical complications, defined as wound infections or dehiscence, intra-abdominal infection or bleeding, rectal suture line dehiscence, fistulas, stoma complications, small bowel obstruction, and the presence of a urinary catheter at discharge. Secondary endpoints included major surgical complication, perineopelvic complications, and the overall complication rate. All complications were categorized according to the Clavien–Dindo classification. Major surgical complications were defined as any surgical complication classified as Clavien–Dindo Grade ≥ IIIa (that is, requiring surgical, endoscopic, or radiological intervention). Perineopelvic complications included perineal wound infections, pelvic abscess or haematoma, rectal staple line insufficiency (including fistula), and the presence of a urinary catheter at discharge. Overall complications were defined as any surgical or medical complications.

### Sample size and statistical analysis

In this study, it was hypothesized that 30-day surgical complications would be reduced by 20% with iAPE compared with HP. To achieve statistical power, a sample size of 140 patients per group would be required with a two-sided α of 0.05 and 1–β of 0.8. Assuming a dropout rate of 20%, the final target enrolment was set at 170 per group. Data were analysed using intention-to-treat (ITT) analyses, with an additional per-protocol analysis also performed. An interim analysis for sample size reassessment was planned after the enrolment of 150 randomized patients, to be performed by three surgeons independently. Descriptive statistics, including continuous variables presented as the mean and standard deviation (s.d.) and categorical variables presented as numbers and percentages, were used to summarize baseline characteristics, surgical details, and complication rates. Logistic regression in the ITT population served as the primary analysis to compare the surgical procedures. For exploratory purposes, univariable and multivariable logistic regression analyses were used to identify potential predictors of complications. These were also used for sensitivity analyses to gauge the potential effect of unbalanced predictors between treatment arms on trial results. Furthermore, sensitivity analyses, including Poisson regression with robust standard errors and mixed-effects logistic regression with hospital as a random effect, were conducted to validate the findings and account for the multicentre design. A per-protocol analysis further evaluated the consistency of results. All statistical analyses were performed using SPSS^®^ version 28 (IBM, Armonk, NY, USA).

## Results

Throughout the enrolment phase, the 15 participating centres collectively randomized 163 patients, with individual centre contributions ranging from 1 to 35 patients. The study was halted prematurely based on recommendations from an independent study committee following a preplanned interim analysis after 150 randomized patients. This was attributed to a lower-than-anticipated inclusion rate and minimal differences in complication rates between the study groups. In all, of 194 eligible patients, 163 patients were randomized, with 80 allocated to the HP group and 83 allocated to the iAPE group (*Fig. 1*).

### Baseline clinical characteristics

Baseline clinical characteristics and reasons for not performing an anterior resection with anastomosis were well balanced across the groups (*Table 1*). In the HP group, 38% of patients were male, compared with 48% in the iAPE group. A higher proportion of patients in the HP group received preoperative radiotherapy (65%) than in the iAPE group (47%). None of the patients received postoperative radiotherapy.

**Table 1:** Baseline clinical characteristics of patients randomized to HP or iAPE

	HP (*n* = 80)	iAPE (*n* = 83)
Age (years), mean(s.d.)	78(8)	79(8)
**Sex**		
Male	30 (38%)	40 (48%)
Female	50 (62%)	43 (52%)
BMI (kg/m^2^), mean(s.d.)	26(4)	25(4)
**Co-morbidity**	58 (73%)	57 (69%)
Missing	7 (9%)	6 (7%)
**Never smoked**	56 (70%)	46 (55%)
Missing	4 (5%)	5 (6%)
WHO performance status 0–1	78 (98%)	79 (95%)
**ASA Grade III**	45 (56%)	44 (53%)
Missing	0	2 (2%)
Tumour distance (cm), mean(s.d.)	9(3)	9(3)
Preoperative radiotherapy	52 (65%)	39 (47%)
Preoperative chemotherapy **cTNM stage**	17 (21%)	17 (20%)
I–II	41 (51%)	47 (57%)
II	25 (31%)	28 (34%)
IV	10 (13%)	3 (4%)
Missing	4 (5%)	5 (6%)
**Reason for not performing LAR**		
Poor sphincter function	18 (23%)	19 (23%)
Age/co-morbidities	44 (55%)	44 (53%)
Other	16 (20%)	15 (18%)
Missing	2 (2%)	5 (6%)

Values are *n* (%) unless otherwise stated. HP, Hartmann’s procedure; iAPE, intersphincteric abdominoperineal excision; s.d., standard deviation; BMI, body mass index; WHO, World Health Organization; ASA, American Society of Anesthesiologists; cTNM, clinical Tumour, Node, and Metastasis staging; LAR, low anterior resection.

### Intraoperative findings

Intraoperative findings (*Table 2*) showed that HP procedures were, on average, 82 minutes shorter than iAPE procedures. Minimally invasive surgery was used in 55% of the HP procedures, compared with 66% of the iAPE procedures, and the overall conversion rate to open surgery was 15%, without any difference between the two groups. Intraoperative bowel perforation occurred in 5% of patients in the HP group and in 8% of patients in the iAPE group. The mean estimated surgical blood loss was 426 ml in the HP group and 316 ml in the iAPE group.

**Table 2:** Operative findings of patients in the HAPIrect trial

	HP (*n* = 80)	iAPE (*n* = 83)
Operating time (minutes), mean(s.d.)	291(105)	373(108)
High ligation of IMA	40 (50%)	33 (40%)
Intraoperative bowel perforation	4 (5%)	7 (8%)
Abdominal pelvic drain	59 (79%)	55 (71%)
Intraoperative bleeding (ml), mean(s.d.)	426(789)	316(457)
Open surgery	34 (43%)	28 (34%)
**Minimally invasive surgery**	44 (55%)	55 (66%)
Laparoscopic	11 (14%)	11 (13%)
Robotic-assisted	33 (41%)	44 (53%)
Conversion to open surgery	6 (14%)	8 (16%)

Values are *n* (%) unless otherwise stated. HP, Hartmann’s procedure; iAPE, intersphincteric abdominoperineal excision; s.d., standard deviation; IMA inferior mesenteric artery.

A local radical excision (R0) was performed in 152 patients (93%); the R status was unknown in 9 (6%) patients. The mean circumferential resection margin was 13 mm in both groups.

### Thirty-day complications

Analyses of 30-day complication rates are presented in *Table 3*. The surgical complication rate in the HP and iAPE groups was 39% and 43%, respectively, with an odds ratio (OR) for HP of 0.83 (95% confidence interval (c.i.) 0.44 to 1.54; *P* = 0.549). The rate of major surgical complications (Clavien–Dindo grade ≥ IIIa) in the HP and iAPE groups was 14% and 11%, respectively (OR for HP 1.31; 95% c.i. 0.51 to 3.36; *P* = 0.573), and the overall complication rate was 45% and 49%, respectively (OR for HP 0.84; 95% c.i. 0.45 to 1.55; *P* =0.574) (*Table 3*).

**Table 3:** Analyses of 30-day postoperative complications after HP and iAPE

	HP (*n* = 80)	iAPE (*n* = 83)	P*
**Surgical complications**	31 (39%)	36 (43%)	
Odds ratio†	0.83 (0.44, 1.54)	1 (reference)	0.549
**Surgical complication CD ≥ IIIa**	11 (14%)	9 (11%)	
Odds ratio†	1.31 (0.51, 3.36)	1 (reference)	0.573
**Overall complications**	36 (45%)	41 (49%)	
Odds ratio†	0.84 (0.45, 1.55)	1 (reference)	0.574
**Perineopelvic complication**	17 (21%)	25 (30%)	
Odds ratio†	0.63 (0.31, 1.28)	1 (reference)	0.197
**Perineopelvic complication CD ≥ IIIa**	7 (9%)	4 (5%)	
Odds ratio†	1.89 (0.53, 6.74)	1 (reference)	0.324

Values are *n* (%) unless otherwise stated. *Univariable logistic regression analysis. †Values in parentheses are 95% confidence intervals. HP, Hartmann’s procedure; iAPE, intersphincteric abdominoperineal excision; CD, Clavien–Dindo.

Twenty patients experienced major surgical complications (Clavien–Dindo grade ≥ III). Laparotomy or laparoscopy was performed in nine patients with extrapelvic complications (such as wound dehiscence, stomal complications, small bowel injury) not related to the pelvic dissection. Eleven patients had major perineopelvic complications that required drainage or surgery.

Perineopelvic complications occurred in 21% and 30% of patients in the HP and iAPE groups, respectively (OR for HP 0.63; 95% c.i. 0.31 to 1.28; *P* = 0.197), with the rate of major perineopelvic complications (Clavien–Dindo grade ≥ IIIa) being 8.8% and 4.6%, respectively (OR for HP 1.89; 95% c.i. 0.53 to 6.74; *P* = 0.324) (*Table 3*). Seven patients (9%) developed pelvic abscesses after HP; all were managed with percutaneous/transanal drainage. After iAPE, four patients (5%) developed major perineopelvic complications: one patient with fistula and one with postoperative bleeding managed with surgery and two patients with a pelvic abscess managed with percutaneous drainage. Neither of the patients with a pelvic abscess had the anorectal stump removed during the 30-day follow-up. No perineal wound breakdown after iAPE was registered.

The 30-day mortality rate was 1.3% in the HP group and 2.4% in the iAPE group, which was not significantly different (*P* = 1.000).

Overall complications (that is, any surgical or medical complications) were registered in 45% of patients in the HP group and in 49% of patients in the iAPE group (OR 0.84, c.i. 0.45–1.55, *P* = 0.574) (*Table 3*). In multivariable regression analysis, which adjusted for differences in sex and preoperative radiotherapy, the OR for surgical complications after HP compared with iAPE was 0.78 (c.i. 0.41–1.50, *P* = 0.463) (*Table 4*). The mean(s.d.) length of hospital stay was 10(7) days in patients who underwent HP and 11(7) days for those who underwent iAPE.

**Table 4:** Multivariable logistic regression analysis of 30-day surgical complications

	Odds ratio∗	*P*
**Sex**		
Female	1 (reference)	
Male	1.00 (0.52, 1.90)	0.988
**Preoperative radiotherapy**		
No	1 (reference)	
Yes	1.18 (0.61, 2.28)	0.616
**Surgical method**		
iAPE	1 (reference)	
HP	0.78 (0.41, 1.50)	0.463

*Values in parentheses are 95% confidence intervals. iAPE, intersphincteric abdominoperineal excision; HP, Hartmann’s procedure.

### Sensitivity analysis

In the sensitivity analysis using a Poisson regression model, which included hospital as a random effect, the surgical method—either HP or iAPE—did not significantly affect surgical complications (*Table S2*). Mixed-effects logistic regression analysis with hospital as a random effect resulted in minor non-significant differences between HP and iAPE (OR 0.79; 95% c.i. 0.41–1.53; *P* = 0.485). The per-protocol analysis revealed no notable differences compared with the ITT analysis because only one patient randomized to iAPE was operated on with HP. In an additional multivariable regression model, including open or minimal invasive surgery had no significant effect on the overall complication rate between the HP and iAPE groups (*Table S3*).

## Discussion

In this multicentre RCT, no clinically significant differences were observed in postoperative surgical complications between HP and iAPE in patients with rectal cancer who were unsuitable for an anastomosis. Similarly, no significant differences were found for the secondary outcomes of overall and perineopelvic complications. Operation time was prolonged by more than 1 hour for iAPE. For the majority of patients, the reason for not performing LAR was advanced age and/or the presence of co-morbidities. The number of patients undergoing minimally invasive surgery was low overall, and slightly lower in patients who underwent HP; however, this difference had no significant impact on the rate of surgical complications between the two groups.

Trial inclusion was stopped following a preplanned interim analysis because of slow accrual and minimal differences in complications between the two groups. Even if the study had reached completion, it is unlikely that any clinically significant difference in surgical complications between the two surgical procedures would have been detected.

The main surgical difference between the two procedures is perineal dissection, which is associated with a unique spectrum of complications. Therefore, an analysis was conducted of perineopelvic complications, defined as complications attributed to pelvic and perineal dissection, which indicated that the surgical trauma and prolonged operating time associated with iAPE resulted in numerically more minor perineopelvic complications. The rate of major (Clavien–Dindo grade ≥ IIIa) perineopelvic complications after HP (that is, those necessitating intervention) was though numerically higher than in iAPE group, but remained low (9%). Furthermore, the decision regarding non-restorative surgery was made before surgery, in contrast with a previous published retrospective study^19^, which reported that an intraoperative decision to perform HP was made in 23% of patients.

The confidence intervals for most of the endpoints in the present study are wide. To confirm whether the statistically non-significant differences in perineopelvic complications observed in this study are clinically significant, a study population exceeding 1300 patients would be required, which is unlikely to be feasible in a RCT.

In a recently published non-randomized registry-based study^17^ encompassing over 1000 patients that used inclusion criteria similar to those of the HAPIrect trial, the risk of surgical complications was lower after iAPE in the adjusted analysis. This may highlight the shortcomings of non-randomized trials in fully adjusting for different confounding factors, although those results are uncertain, and some differences may be due to random variation. It is important to note that in the present study both surgical procedures were viable options, and randomization was performed during surgery after clamping distal to the tumour.

Even though the HAPIrect trial used intraoperative randomization to mitigate potential confounders, the HP group had significantly more patients who had received preoperative radiotherapy. To account for this, logistic regression analysis and sensitivity analysis were performed, both of which confirmed that differences in surgical complications between the two procedures were small in this study population.

In the HAPIrect trial, high age and co-morbidities were stated as the reason for non-restorative surgery in 60% of patients. Furthermore, one-quarter of patients were included because of impaired anal sphincter function. Given the absence of clear distinctions between the HP and iAPE groups concerning surgical complications, the oncological long-term results and quality of life become even more important for optimizing surgical care in this aging, co-morbid population. The finding of a 6.7% rate of intraoperative bowel perforations after iAPE in the present study is of concern and aligns with previous reports^16,17^. High age, co-morbidities, and complications, together with ostomy training, contributed to a longer length of hospital stay in both groups.

A limitation of the HAPIrect trail was the low inclusion rate, resulting in an extended inclusion period, with several hospitals enrolling fewer than ten patients during the inclusion period. The relatively low number of patients randomized further contributed to the study’s limitations. One potential explanation for this phenomenon is operating surgeons’ preconceived opinions regarding the preferred procedure to be performed. This also affected the registration of patients in the CONSORT flowchart who were not asked to participate. Despite efforts, systematic errors in randomization could not be identified. The differences in patient characteristics between the groups were adjusted for in the sensitivity analyses, without notably affecting the results. It should also be noted that the results are representative of patients with a rectal tumour located ≥ 5 cm from the anal verge.

In conclusion, both HP and iAPE were viable surgical options for patients with rectal cancer who are unsuitable for anastomosis in this RCT. In the absence of long-term data on long-term complications, quality of life, and oncological outcomes, it is crucial to emphasize that the most important factor is that surgeons, in shared decision-making with the patient, prioritize the operative technique with which they are most confident and experienced.

## Collaborators

Eva Angenete (Department of Surgery, Sahlgrenska University Hospital, Region Västra Götaland, Gothenburg, Sweden); Helgi Birgisson (Department of Surgery, Institution of Surgical Sciences, Uppsala University, Uppsala, Sweden); Abbas Chabok (Division of Surgery, Danderyd University Hospital, Stockholm, Sweden); George Dafnis (Department of Surgery and Urology, Eskilstuna County Hospital, Eskilstuna, Sweden); Markku Haapamäki (Department of Surgical and Perioperative Sciences, Surgery, Umeå University, Umeå, Sweden); Peter Matthiessen (Department of Surgery, Faculty of Medicine and Health Sciences, Örebro University, Örebro, Sweden); Pär Myrelid (Department of Surgery, Linköping University Hospital, Linköping, Sweden); Gert Nestler (Department of Surgery, Falun Hospital, Region Dalarna, Sweden); Christoffer Odensten (Department of Surgery and Perioperative Sciences, Umeå University, Umeå, Sweden); Jukka Rintala (Department of Surgery, Oulu University Hospital, Oulu, Finland); Thorbjörn Sakari (Department of Surgery, CFUG, Gävle Hospital, Gävle, Sweden); Josefin Segelman (Department of Molecular Medicine and Surgery, Karolinska Institutet, Stockholm, Sweden; Department of Surgery, Ersta Hospital, Stockholm, Sweden); Ingvar Sverrisson (Department of Surgery, Västmanland's Hospital Västerås, Västerås, Sweden); Niklas Zar (Department of Surgery, Ryhov County Hospital, Jönköping, Sweden); Philippe Wagner (Centre for Clinical Research Region, Västmanland Uppsala University, Västerås, Sweden)

## Supplementary Material

zraf093_Supplementary_Data

## Data Availability

The study data are available from the corresponding author on reasonable request.

## References

[1] Heald RJ, Moran BJ, Ryall RD, Sexton R, MacFarlane JK. Rectal cancer: the Basingstoke experience of total mesorectal excision, 1978–1997. Arch Surg 1998;133:894–899 9711965 10.1001/archsurg.133.8.894

[2] Emmertsen KJ, Laurberg S. Low anterior resection syndrome score: development and validation of a symptom-based scoring system for bowel dysfunction after low anterior resection for rectal cancer. Ann Surg 2012;255:922–928 22504191 10.1097/SLA.0b013e31824f1c21

[3] Påhlman L . Svenska Kolorektalcancerregistret (Ändtarm). 2024. https://statistik.incanet.se/kolorektal/rektum/ (accessed 15 January 2024)

[4] Hosseinali Khani M, Pahlman L, Smedh K. Treatment strategies for patients with stage IV rectal cancer: a report from the Swedish Rectal Cancer Registry. Eur J Cancer 2012;48:1616–1623 22306019 10.1016/j.ejca.2011.12.012

[5] Påhlman L, Bohe M, Cedermark B, Dahlberg M, Lindmark G, Sjödahl R et al The Swedish Rectal Cancer Registry. Br J Surg 2007;94:1285–1292 17661309 10.1002/bjs.5679

[6] Meyer F, Marusch F, Koch A, Meyer L, Führer S, Köckerling F et al Emergency operation in carcinomas of the left colon: value of Hartmann's procedure. Tech Coloproctol 2004;8 (Suppl 1):s226–s229 15655630 10.1007/s10151-004-0164-3

[7] Heah SM, Eu KW, Ho YH, Leong AF, Seow-Choen F. Hartmann's procedure vs. abdominoperineal resection for palliation of advanced low rectal cancer. Dis Colon Rectum 1997;40:1313–1317 9369105 10.1007/BF02050815

[8] Mariusdottir E, Jörgren F, Mondlane A, Wikström J, Lydrup ML, Buchwald P. Low incidence of pelvic sepsis following Hartmann's procedure for rectal cancer: a retrospective multicentre study. BMC Surg 2022;22:421 36494661 10.1186/s12893-022-01858-8 PMC9733326

[9] Sverrisson I, Nikberg M, Chabok A, Smedh K. Low risk of intra-abdominal infections in rectal cancer patients treated with Hartmann's procedure: a report from a national registry. Int J Colorectal Dis 2018;33:327–332 29354849 10.1007/s00384-018-2967-0 PMC5816765

[10] Sverrisson I, Nikberg M, Chabok A, Smedh K. Hartmann's procedure in rectal cancer: a population-based study of postoperative complications. Int J Colorectal Dis 2015;30:181–186 25421100 10.1007/s00384-014-2069-6

[11] Tottrup A, Frost L. Pelvic sepsis after extended Hartmann's procedure. Dis Colon Rectum 2005;48:251–255 15714249 10.1007/s10350-004-0767-9

[12] Molina Rodríguez JL, Flor-Lorente B, Frasson M, García-Botello S, Esclapez P, Espí A et al Low rectal cancer: abdominoperineal resection or low Hartmann resection? A postoperative outcome analysis. Dis Colon Rectum 2011;54:958–962 21730783 10.1097/DCR.0b013e31821c4b95

[13] Frye JN, Carne PW, Robertson GM, Frizelle FA. Abdominoperineal resection or low Hartmann's procedure. ANZ J Surg 2004;74:537–540 15230785 10.1111/j.1445-2197.2004.03055.x

[14] Ahmad NZ, Azam M, Coffey JC. A meta-analysis of low Hartmann's procedure *versus* abdominoperineal resection for non-restorative treatment of rectal cancer. Int J Colorectal Dis 2021;36:2585–2598 34272997 10.1007/s00384-021-03993-9

[15] Choy KT, Lee DJ, Prabhakaran S, Warrier S, Heriot A, Kong JC. The complication profile of low Hartmann's in rectal cancer: a systematic review and meta-analysis. ANZ J Surg 2022;92:2829–2839 35727062 10.1111/ans.17827

[16] Fowler H, Clifford R, Sutton P, Watson A, Fearnhead N, Bach S et al Hartmann's procedure *versus* intersphincteric abdominoperineal excision (HiP study): a multicentre prospective cohort study. Colorectal Dis 2020;22:2114–2122 32939956 10.1111/codi.15366

[17] Åkerlund V, Nikberg M, Wagner P, Chabok A. Hartmann’s procedure *versus* intersphincteric abdominoperineal excision in patients with rectal cancer: report from the Swedish Colorectal Cancer Registry (SCRCR). Ann Surg Open 2024;5:e428 38911665 10.1097/AS9.0000000000000428 PMC11191996

[18] Smedh K, Sverrisson I, Chabok A, Nikberg M; HAPIrect Collaborative Study Group. Hartmann's procedure vs abdominoperineal resection with intersphincteric dissection in patients with rectal cancer: a randomized multicentre trial (HAPIrect). BMC Surg 2016;16:43 27401339 10.1186/s12893-016-0161-2 PMC4940760

[19] Mariusdottir E, Jörgren F, Saeed M, Wikström J, Lydrup ML, Buchwald P. Hartmann's procedure in rectal cancer surgery is often an intraoperative decision: a retrospective multicenter study. Langenbecks Arch Surg 2024;409:55 38321307 10.1007/s00423-024-03237-8 PMC10847187

