# Supplementary material for: Postoperative complications in Hartmann’s procedure versus intersphincteric abdominoperineal excision in rectal cancer: randomized clinical trial (HAPIrect)
Source: BJS Open. 2025 Sep 30;9(5):zraf093. doi: 10.1093/bjsopen/zraf093 (PMC12482908; doi:10.1093/bjsopen/zraf093)
Supplement: zraf093_Supplementary_Data [file zraf093_supplementary_data.zip › Supplementary_Material.docx]

**Postoperative complications in Hartmann’s procedure versus intersphincteric abdominoperineal excision in rectal cancer: randomized controlled trial (HAPIrect**)

Maziar Nikberg MD PhD^1,2^, Viktor Åkerlund MD^1,2^, Torbjörn Swartling MD PhD^3^, Pamela Buchwald MD PhD^4^, Kenneth Smedh MD PhD^1,2^

HAPIrect Collaborative Study Group

^1^ Department of Surgery, Västmanland's hospital Västerås, Sweden

^2^ Centre for Clinical Research Region Västmanland, Uppsala University, Sweden

^3^ Department of Surgery, Institute of Clinical Sciences, Sahlgrenska Academy, University of Gothenburg, Sahlgrenska University Hospital/Östra, Gothenburg, Sweden

^4^ Department of Surgery, Skåne University Hospital, Malmö, Lund University, Lund, Sweden

**Corresponding author.** Name and address **ORCID ID**; **Twitter**

Maziar Nikberg, Department of Surgery, Västmanland´s Hospital Västerås, 721 89 Västerås, Sweden. Phone: +46 70-418 57 21

[maziar.nikberg@regionvastmanland.se](mailto:maziar.nikberg@regionvastmanland.se)

ORCID: 0000-0002-5949-3810

**Supplementary Materials - Index**

| **Supplementary Appendixes** |  |
| --- | --- |
| HAPIrect Collaborative Study Group | *page 2* |
| **Supplementary Figures and Tables** |  |
| supplementary Table1 | *page 3* |
| supplementary Table 2 | *page 4* |
| supplementary Table 3 | *page 5* |
| **References** | *page 6* |

**HAPIrect Collaborative Study Group (All to be PubMed citable)**

Eva Angenete (Department of Surgery, Sahlgrenska University Hospital, Region Västra Götaland, Gothenburg, Sweden); Helgi Birgisson (Department of Surgery, Institution of Surgical Sciences, Uppsala University, Uppsala, Sweden); Abbas Chabok (Division of Surgery, Danderyd University Hospital, Stockholm, Sweden); George Dafnis (Department of Surgery and Urology, Eskilstuna County Hospital, Eskilstuna, Sweden); Markku Haapamäki (Department of Surgical and Perioperative Sciences, Surgery, Umeå University, Umeå, Sweden); Peter Matthiessen (Department of Surgery, Faculty of Medicine and Health Sciences, Örebro University, Örebro, Sweden); Pär Myrelid (Department of Surgery, Linköping University Hospital, Linköping, Sweden); Gert Nestler (Department of Surgery, Falun Hospital, Region Dalarna, Sweden); Christoffer Odensten (department of surgery and perioperative sciences, Umeå University, Sunderby Research Unit, Sweden); Jukka Rintala (Department of Surgery, Oulu University Hospital, Oulu, Finland); Thorbjörn Sakari (Department of Surgery, CFUG, Gävle Hospital, Gävle, Sweden); Josefin Segelman (Department of Molecular Medicine and Surgery, Karolinska Institutet, and Department of Surgery, Ersta Hospital, Stockholm, Sweden); Ingvar Sverrisson (Department of Surgery, Västmanland's hospital Västerås, Sweden); Niklas Zar (Department of Surgery , Ryhov County Hospital , Jönköping , Sweden); Philippe Wagner (Centre for Clinical Research Region, Västmanland Uppsala University, Sweden)

**Supplementary Table 1.**

Inclusion and exclusion criteria in HAPIrect trail

| **Inclusion criteria** | **Exclusion criteria** |
| --- | --- |
| Age 18 years or older  Rectal adenocarcinoma within 5 to 15 cm from the anal verge  Anterior resection with anastomosis deemed unsuitable  Both Hartman’s procedure and intersphincteric abdominoperineal excision possible  Surgery may be palliative but should aim for a locally radical resection (R0) | Rectal cancer below 5 cm from the anal verge |
|  | ASA IV/V  Non-correctable coagulopathy |
|  |  |

**Supplementary Table 2.** Poisson regression model of 30-day surgical complications

|  | Relative Risk | 95 % CI | *P* |
| --- | --- | --- | --- |
| Gender  Female  Male | 1  1.02 | 0.28–1.48 | 0.929 |
| Preoperative radiotherapy  No  Yes | 1  1.13 | 0.77–1.66 | 0.524 |
| Surgical method  iAPE  HP | 1  0.88 | 0.60–1.28 | 0.492 |

HP Hartmann’s procedure; iAPE intersphincteric abdominoperineal excision.

**Supplementary Table 3.** Multivariable logistic regression analysis of 30-day surgical complications

|  | Relative Risk | 95 % CI | *P* |
| --- | --- | --- | --- |
| Gender  Female  Male | 1  1.08 | 0.56–2.08 | 0.828 |
| Preoperative radiotherapy  No  Yes | 1  1.12 | 0.58–2.19 | 0.731 |
| Surgical approach  Open  MIS | 1  0.53 | 0.27–1.03 | 0.062 |
| Surgical method  iAPE  HP | 1  0.78 | 0.40–1.51 | 0.459 |

MIS minimal invasive surgery (laparoscopic/robotic surgery); HP Hartmann’s procedure; iAPE intersphincteric abdominoperineal excision.

**References**

1. Heald RJ, Moran BJ, Ryall RD, et al. Rectal cancer: the Basingstoke experience of total mesorectal excision, 1978-1997. *Arch Surg* 1998; 133(8):894-9.

2. Emmertsen KJ, Laurberg S. Low anterior resection syndrome score: development and validation of a symptom-based scoring system for bowel dysfunction after low anterior resection for rectal cancer. *Ann Surg* 2012; 255(5):922-8.

3. Svenska Kolorektalcancerregistret (Ändtarm). [Internet]. Stockholm: Regionala cancercentrum i samverkan; 2024. [cited 2024-01-15 from <https://statistik.incanet.se/kolorektal/rektum/>] [database online].

4. Hosseinali Khani M, Pahlman L, Smedh K. Treatment strategies for patients with stage IV rectal cancer: a report from the Swedish Rectal Cancer Registry. *Eur J Cancer* 2012; 48(11):1616-23.

5. Pahlman L, Bohe M, Cedermark B, et al. The Swedish rectal cancer registry. *Br J Surg* 2007; 94(10):1285-92.

6. Meyer F, Marusch F, Koch A, et al. Emergency operation in carcinomas of the left colon: value of Hartmann's procedure. *Tech Coloproctol* 2004; 8 Suppl 1:s226-9.

7. Heah SM, Eu KW, Ho YH, et al. Hartmann's procedure vs. abdominoperineal resection for palliation of advanced low rectal cancer. *Dis Colon Rectum* 1997; 40(11):1313-7.

8. Mariusdottir E, Jorgren F, Mondlane A, et al. Low incidence of pelvic sepsis following Hartmann's procedure for rectal cancer: a retrospective multicentre study. *BMC Surg* 2022; 22(1):421.

9. Sverrisson I, Nikberg M, Chabok A, et al. Low risk of intra-abdominal infections in rectal cancer patients treated with Hartmann's procedure: a report from a national registry. *Int J Colorectal Dis* 2018; 33(3):327-332.

10. Sverrisson I, Nikberg M, Chabok A, et al. Hartmann's procedure in rectal cancer: a population-based study of postoperative complications. *Int J Colorectal Dis* 2015; 30(2):181-6.

11. Tottrup A, Frost L. Pelvic sepsis after extended Hartmann's procedure. *Dis Colon Rectum* 2005; 48(2):251-5.

12. Molina Rodriguez JL, Flor-Lorente B, Frasson M, et al. Low rectal cancer: abdominoperineal resection or low Hartmann resection? A postoperative outcome analysis. *Dis Colon Rectum* 2011; 54(8):958-62.

13. Frye JN, Carne PW, Robertson GM, et al. Abdominoperineal resection or low Hartmann's procedure. *ANZ J Surg* 2004; 74(7):537-40.

14. Ahmad NZ, Azam M, Coffey JC. A meta-analysis of low Hartmann's procedure versus abdominoperineal resection for non-restorative treatment of rectal cancer. *Int J Colorectal Dis* 2021; 36(12):2585-2598.

15. Choy KT, Lee DJ, Prabhakaran S, et al. The complication profile of low Hartmann's in rectal cancer: a systematic review and meta-analysis. *ANZ J Surg* 2022; 92(11):2829-2839.

16. Fowler H, Clifford R, Sutton P, et al. Hartmann's procedure versus intersphincteric abdominoperineal excision (HiP Study): a multicentre prospective cohort study. *Colorectal Dis* 2020; 22(12):2114-2122.

17. Åkerlund V, Nikberg M, Wagner P, et al. Hartmann’s Procedure Versus Intersphincteric Abdominoperineal Excision in Patients with Rectal Cancer: Report from the Swedish Colorectal Cancer Registry (SCRCR). *Annals of Surgery Open* 2024; 5(2):e428.

18. Smedh K, Sverrisson I, Chabok A, et al. Hartmann's procedure vs abdominoperineal resection with intersphincteric dissection in patients with rectal cancer: a randomized multicentre trial (HAPIrect). *BMC Surg* 2016; 16(1):43.

19. Mariusdottir E, Jörgren F, Saeed M, et al. Hartmann's procedure in rectal cancer surgery is often an intraoperative decision: a retrospective multicenter study. *Langenbecks Arch Surg* 2024; 409(1):55.
